# Suicide in adolescents: findings from the Swiss National cohort

**DOI:** 10.1007/s00787-017-1019-6

**Published:** 2017-06-29

**Authors:** Nicole Steck, Matthias Egger, Benno G. Schimmelmann, Stephan Kupferschmid, for the Swiss National Cohort

**Affiliations:** 1 Institute of Social and Preventive Medicine, University of Bern, Finkenhubelweg 11, 3012 Bern, Switzerland; 2 Department of Child- and Adolescent Psychiatry, University of Bern, Bern, Switzerland; 3 University Hospital of Child and Adolescent Psychiatry, University Hospital Hamburg Eppendorf, Hamburg, Germany; 4 Department of Child and Adolescent Psychiatry and Psychotherapy, Psychiatric Services Aargau (Teaching Hospital of the University of Zurich), Brugg, Switzerland

**Keywords:** Suicide, Adolescents, Children, Switzerland, Longitudinal study

## Abstract

Suicide in adolescents is the second most common cause of death in this age group and an important public health problem. We examined sociodemographic factors associated with suicide in Swiss adolescents and analysed time trends in youth suicide in the Swiss National Cohort (SNC). The SNC is a longitudinal study of the whole Swiss resident population, based on linkage of census and mortality records. We identified suicides in adolescents aged 10–18 years from 1991 to 2013. A total of 2.396 million adolescents were included and 592 suicides were recorded, corresponding to a rate of 3.7 per 100,000 [95% confidence interval (CI) 3.4–4.0]. Rates increased with age from 0.0 per 100,000 at age 10 years to 14.8 per 100,000 (95% CI 12.6–17.5) at 18 years in boys, and from 0.0 to 5.4 per 100,000 (4.1–7.2) in girls. Being a boy, living in a single parent household, being an only or middle-born child, and living in rural regions were factors associated with a higher rate of suicide. Hanging was the most common method in boys, and railway suicides were most frequent in girls. There was no clear evidence for an increase or decrease over calendar time. We conclude that familial and socioeconomic factors including type of household, birth order and urbanity are associated with youth suicide in Switzerland. These factors should be considered when designing prevention programmes for youth suicide.

**Electronic supplementary material:**

The online version of this article (doi:10.1007/s00787-017-1019-6) contains supplementary material, which is available to authorized users.

## Introduction

Recognised as an important public health problem by the World Health Organization (WHO) [1], suicide in youth is a rare but tragic event, with severe implications for the families and friends concerned. Suicide rates increase steeply from childhood to adolescence [2, 3]. In adolescents, suicide is the second most common cause of death [4], and up to 30% of all adolescents have suicidal thoughts or behaviours [5].

Mental disorders, particularly depressive disorders, anxiety disorders and substance use, are associated with suicide and suicidal behaviour not only in adults, but also in adolescents [2–4, 6, 7]. Adolescent girls experience a higher rate of suicidal ideation and suicide attempts [6, 8], but boys are more likely to complete suicide than girls [3, 6, 8] mirroring the situation in adults. Familial and socioeconomic factors have also been identified as risk factors for youth suicide [3, 9]: parental psychopathology [6, 7, 10], a family history of suicidal behaviour [6, 7], being born to young mothers [11, 12] and mothers with a low education [12], parental divorce [2, 10], living in single parent households [7, 13] and parent–child conflicts [7, 14]. Studies from Norway and Scotland found that a higher position in birth order was positively associated with the risk for suicide, but both studies included not only adolescents, but also young adults [11, 15]. Whereas conflicts with parents and maltreatment are often reported in suicides of younger adolescents (typically defined as ages 10–15 years), crises in romantic relationships and psychological disorders are more important in older adolescents [16–18]. There are only few data on time trends in suicides in youths, and results are inconsistent across studies [19, 20]. Analysing the data of the WHO for Europe, Kolves et al. found a decrease in suicide rates in age group 10–14 years and in age group 15–19 years old, although in the younger age group no decrease was found in girls [21, 22].

We analysed a large national cohort study to examine suicide rates in youth, the chosen methods of suicide and reported psychiatric conditions. Our aim was to investigate sociodemographic factors associated with suicide in adolescents in Switzerland and to examine trends in suicide rates over time.

## Methods

### The Swiss National Cohort

The Swiss National Cohort (SNC) is a longitudinal study of mortality in Switzerland based on linkage of census and mortality records. The SNC and details regarding the linkage process have been described in detail elsewhere [23–25]. Briefly, the records of the 1990 and 2000 censuses were linked to death records or emigration records up to 2013 using deterministic and probabilistic linkage procedures, based on sex, date of birth, place of residence and other variables [23, 24]. The census was mandatory, with population coverage estimated at 98.6% [26]. The SNC was initiated by public health and demography institutes of the Swiss universities Bern, Zurich, Basel, Lausanne and Geneva. The Cantonal Ethics Committees of Bern and Zurich approved the SNC, covering the present analysis. The SNC database is open to national and international researchers. However, access to the data has to be approved by the owner of the data, the Swiss Federal Statistical Office.

### Study population and follow-up

We included all adolescents aged 10–18 years who were registered in the 1990 or 2000 census. In Switzerland adolescents attain the age of majority at 18 years. Follow-up started either on 4 December 1990 (the date of the 1990 census), 5 December 2000 (the date of the 2000 census) or, if a child turned 10 during the study period, on the date of the 10th birthday. We followed all adolescents to the earliest of the 19th birthday, emigration or end of the study period (31 December 2008). Biological mothers and fathers of the adolescents were identified based on the household data and the question in the census asking whether or not adults lived with their own children.

### Identification of suicides

Suicides were identified based on the causes of death recorded on the death certificate. During 1991–1994, deaths caused by intentional self-harm were coded according to the International Classification of Diseases, Eighth Revision (ICD-8), and from 1995 based on the Tenth Revision (ICD-10). We included the following methods of suicides: (1) poisoning (ICD-8 950-952, ICD-10 X60–X69); (2) hanging (ICD-8 953, ICD-10 X70); (3) drowning (ICD-8 954, ICD-10 X71); (4) firearms (ICD-8 955, ICD-10 X72–X75); (5) cutting (ICD-8 956, ICD-10 X78); (6) jumping (ICD-8 957, ICD-10 X80); (7) railway (ICD-8 958.00, ICD-10 X81–X82); other (ICD-8 958 with exception of 958.00, ICD-10 83-84). We also analysed the psychiatric diagnoses reported on the death certificates.

### Statistical analysis

We calculated crude rates by dividing the number of suicides by the number of person-years at risk and calculated univariate and multivariable hazard ratios using Cox regression, with age as the time scale. We included sex, type of household (single person with children, couple with children, institution), birth order (only child, firstborn, middle born or lastborn), highest education of parents (compulsory schooling, secondary, tertiary education), age of mother at birth (in 10-year bands), religion (Protestant, Catholic and no affiliation) and nationality of the adolescent (Swiss or foreign), degree of urbanization of place of residence (urban, peri-urban and rural), socioeconomic position of neighbourhood of residence (quartiles of the Swiss index [27]) and language region (German, French and Italian). The type of household and marital status of parents were strongly correlated, and we therefore did not include marital status. The age of the father was frequently missing and was also not included in the multivariable models. The variables used for analysis originate from census 1990 and census 2000, except for date of death, cause of death and concomitant diseases which were obtained from the death certificates.

Previous studies [2, 6, 28] showed that suicide is very uncommon in childhood and early adolescents and that the rate increases from the age of 15 years. We therefore also examined interactions between age groups (10–14 and 15–18 years) and the socioeconomic factors associated with suicide. In other words, we were interested in any differences in associations between younger and older adolescents.

Statistical analyses were done in Stata version 14 (Stata Corporation, College Station, TX, USA). Results are given as rates per 100,000 population and hazard ratios (HR), with 95% confidence intervals (CI).

## Results

### Study population

A total of 2,395,677 adolescents contributed follow-up time. The characteristic of the study population is shown in Table 1. There were slightly more boys than girls (51 versus 49%). Most adolescents lived with two adults, and most were either first- or lastborn, reflecting the typical number of two children in Swiss families. One-fifth of the adolescents were only children and fewer than 10% had more than two sisters or brothers. The mothers of 6.7% of the adolescents and the fathers of 13.5% could not be identified in the census. More than half of the adolescents without identified father lived only with one adult, compared to fewer than 10% overall. Most mothers were between 25 and 34 years old at birth of the adolescent; 2.8% of adolescents had teenage mothers. In about half of households secondary education was the highest educational level, and about 75% of households were from urban or peri-urban regions.

**Table 1:** Characteristics of adolescents aged 10–18 years, number of suicides, rates of suicide and hazard ratios from univariable Cox models, Switzerland 1991–2013

Characteristic	Number	No. of suicides	Rate per 100,000 (95% CI)	Hazard ratio (95% CI)	*p*
Overall	2,395,677	592	3.7 (3.4–4.0)		
Age (years)					
10–14	2,014,971	75	0.86 (0.68–1.1)	1	
15–18	2,211,573	517	7.0 (6.4–7.6)	6.6* (5.2–8.4)	
Sex					
Female	1,166,443	169	2.1 (1.8–2.4)	1	<0.001
Male	1,229,234	423	5.1 (4.6–5.6)	2.4 (2.1–3.1)	
Type of household					
Couple w. children	2,122,914	489	3.4 (3.1–3.7)	1	0.0214
Single w. children	195,162	82	5.5 (4.4–6.8)	1.4 (1.1–1.7)	
Institution	47,736	10	4.4 (2.4–8.1)	0.94 (0.50–1.8)	
Unknown	29,865	11	8.6 (4.8–15.6)	1.8 (1.0–3.3)	
Birth order					
Only child	474,853	97	4.0 (3.3–4.9)	1.3 (1.02–1.7)	0.062
First born	746,330	167	3.1 (2.7–3.6)	1	
Middle born	264,213	88	4.3 (3.4–5.3)	1.4 (1.05–1.8)	
Last born	750,299	191	3.6 (3.1–4.1)	1.2 (0.94–1.4)	
Unknown	159,982	49	5.1 (3.8–6.7)	1.3 (0.97–1.8)	
Highest education in household					
Compulsory	324,759	59	3.1 (2.4–4.0)	1	0.211
Secondary	1,169,455	291	3.7 (3.3–4.1)	1.3 (0.98–1.7)	
Tertiary	715,677	193	3.7 (3.2–4.3)	1.3 (1.0–1.8)	
Not known	185,786	49	4.3 (3.3–5.7)	1.3 (0.92–2.0)	
Age of mother at birth (years)					
15–24	549,827	142	4.1 (3.4–4.8)	1.1 (0.89–1.3)	0.416
25–34	1,460,535	343	3.4 (3.0–3.8)	1	
35–44	223,793	58	3.9 (3.0–5.0)	1.2 (0.92–1.6)	
Unknown	161,522	49	5.1 (3.8–6.7)	1.2 (0.88–1.6)	
Marital status of mother at census					
Single	50,101	12	4.0 (2.2–7.0)	1.5 (0.82–2.6)	0.260
Married	2,064,253	479	3.4 (3.1–3.8)	1	
Widowed	20,989	8	6.0 (3.0–11.9)	1.3 (0.65–2.6)	
Divorced	100,352	44	5.4 (4.0–7.3)	1.3 (0.96–1.8)	
Unknown	159,982	49	5.1 (3.8–6.7)	1.2 (0.88–1.6)	
Age of father at birth (years)					
15–24	211,660	38	3.0 (2.2–4.1)	0.76 (0.54–1.1)	0.0876
25–34	1,368,964	331	3.6 (3.2–4.0)	1	
35–44	448,334	94	3.1 (2.5–3.8)	0.93 (0.74–1.2)	
≥45	42,659	8	3.0 (1.5–6.0)	0.89 (0.44–1.8)	
Unknown	323,581	121	5.2 (4.3–6.2)	1.2 (0.99–1.5)	
Marital status of father at census					
Single	29,431	4	2.5 (0.95–6.7)	0.98 (0.36–2.6)	0.145
Married	2,014,473	457	3.4 (3.1–3.7)	1	
Widowed	5415	1	2.9 (0.41–20.8)	0.64 (0.09–4.6)	
Divorced	23,472	9	6.1 (3.2–11.8)	1.5 (0.79–2.9)	
Unknown	322,886	121	5.2 (4.3–6.2)	1.3 (1.0–1.6)	
Religion					
Protestant	839,871	231	4.0 (3.5–4.5)	1	0.039
Catholic	1,051,571	262	3.8 (3.3–4.2)	0.94 (0.79–1.1)	
No affiliation	199,995	50	3.7 (2.8–4.8)	1.0 (0.74–1.4)	
Other/unknown	304,240	49	2.5 (1.9–3.3)	0.66 (0.48–0.89)	
Nationality					
Swiss	1,843,429	506	4.0 (3.6–4.3)	1	<0.001
Other	552,248	86	2.6 (2.1–3.2)	0.66 (0.52–0.83)	
Language region					
German	1,714,927	441	3.8 (3.5–4.2)	1	0.416
French	575,880	131	3.4 (2.9–4.1)	0.91 (0.75–1.1)	
Italian	94,893	17	2.7 (1.7–4.4)	0.71 (0.44–1.2)	
Rhaeto-Romance	9977	3	4.3 (1.4–13.3)	1.14 (0.37–3.5)	
Urbanization					
Urban	580,869	112	3.0 (2.5–3.6)	1	<0.001
Peri-Urban	1,089,712	251	3.4 (3.0–3.9)	1.1 (0.91–1.4)	
Rural	725,096	229	4.6 (4.0–5.2)	1.5 (1.2–1.9)	
Neighbourhood index of SEP (quartiles)					
Lowest	600,239	139	3.5 (3.0–4.2)	1	0.943
Second	587,079	144	3.7 (3.1–4.3)	1.0 (0.82–1.3)	
Third	582,101	148	3.7 (3.2–4.4)	1.1 (0.84–1.3)	
Highest	580,303	151	3.8 (3.2–4.5)	1.1 (0.85–1.3)	
Unknown	45,955	10	3.1 (1.7–5.9)	0.86 (0.46–1.6)	

*SEP* socioeconomic position

* Risk ratio (hazard ratio not applicable)

### Rates of suicide

**Fig. 1:**
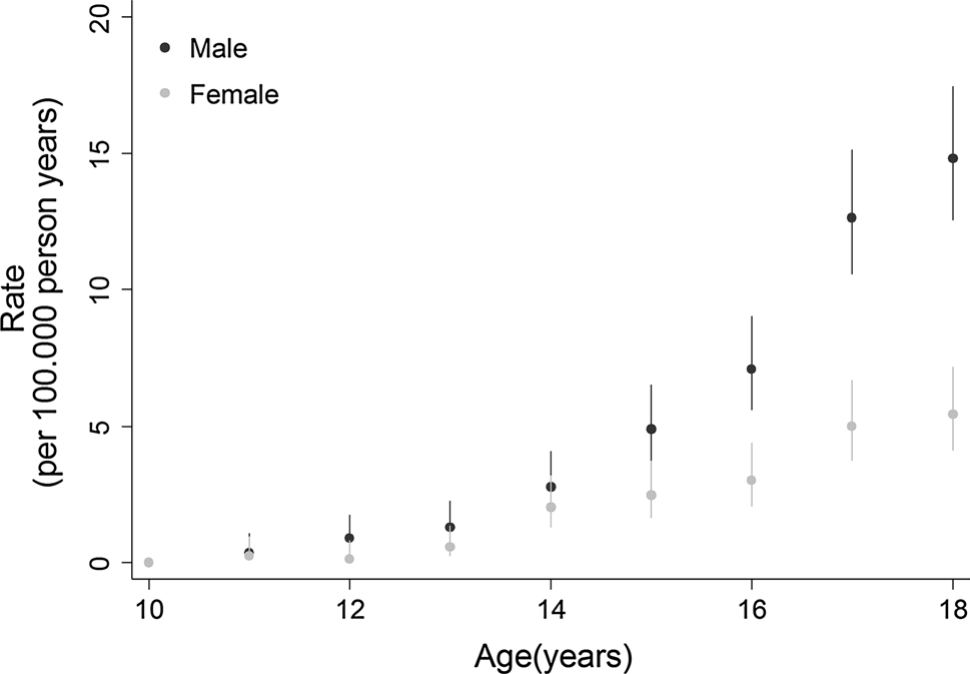
Crude rates of suicide per 100,000 person-years by age and gender in Switzerland, 1991–2013

**Fig. 2:**
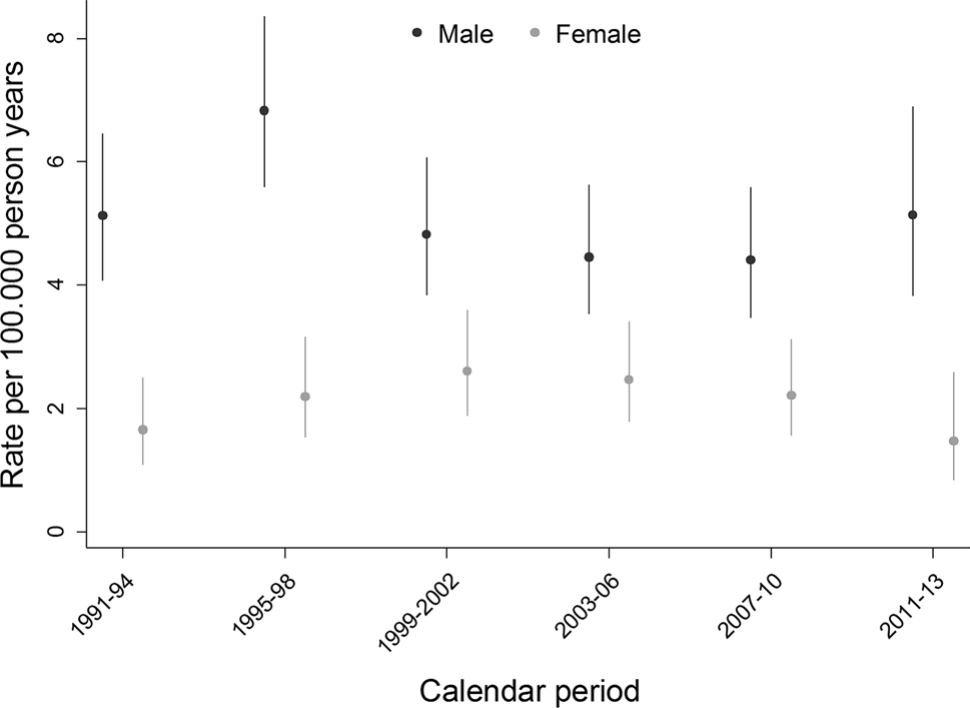
Crude rate of suicide per 100.000 person-years by gender and calendar years in Switzerland, 1991–2013

592 suicides were recorded in adolescents, during 8,738,853 person-years of follow-up. The crude rate of suicide was 3.7 per 100.000 person-years (95% CI 3.4–4.0) (Table 1). Rates increased with age from 0.0 per 100,000 at age 10 years to 14.8 per 100,000 (95% CI 12.6–17.5) at 18 years in boys, and from 0.0 to 5.4 per 100,000 (95% CI 4.1–7.2) in girls (Fig. 1). The rates increased slightly more steeply in boys than in girls and diverged after the age of 14. In boys rates ranged from 4.4 per 100,000 person-years in the period 2007–2010 to 6.8 per 100,000 person-years in 1995–1999 and in girls from 1.47 in 2011–2013 to 2.47 in 1999–2002, with overlapping 95% CIs (Fig. 2).

### Suicide methods and psychiatric diagnoses

Suicide by hanging was the most common method overall, with 159 (26.9%) of 592 suicides (Table 2). Hanging was also the most common method in boys (122 or 28.8%) whereas in girls railway suicides dominated (52 or 30.8%). In boys younger than 15 almost 40% of the 100 suicides were by hanging. In boys but not in girls, suicide by firearms was also a common method (95 or 22.5%). A total of 119 (20.1%) of the 592 suicides had a psychiatric diagnosis on the death certificate (Table 3). Depressive disorders were most frequently reported (74 suicides), followed by alcohol and drug use (13 suicides) and schizophrenia (nine suicides).

**Table 2:** Suicide methods by age group and gender in Switzerland, 1991–2013

	Male	Female	Total
Poisoning	15 (3.5%)	25 (14.8%)	40 (6.8%)
Hanging	122 (28.8%)	37 (21.9%)	159 (26.9%)
Drowning	8 (1.9%)	4 (2.4%)	12 (2.0%)
Firearms	95 (22.5%)	11 (6.5%)	106 (17.9%)
Jumping from height	69 (16.3%)	34 (20.1%)	103 (17.4%)
Railway	104 (24.6%)	52 (30.8%)	156 (26.4%)
Other	10 (2.4%)	6 (3.6%)	16 (2.7%)
Total	423 (100%)	169 (100%)	592 (100%)

Number of suicides (%) are shown

**Table 3:** Mental comorbidities reported on the death certificate of suicides among adolescents, Switzerland 1991–2013

Diagnoses	Number of suicides				
Age at death	10–14 years		15–18 years		Total
	Male	Female	Male	Female	
Depressive episodes	4 (8.2%)	2 (7.7%)	39 (10.4%)	29 (20.3%)	74 (12.5%)
Schizophrenia	–	–	6 (1.6%)	3 (2.1%)	9 (1.5%)
Alcohol and drug use	–	–	7 (1.9%)	6 (4.2%)	13 (2.2%)
Other	3 (6.1%)	3 (11.5%)	13 (3.5%)	4 (2.8%)	23 (3.9%)
All mental disorders	7 (14.3%)	5 (19.2%)	65 (17.4%)	42 (29.4%)	119 (20.1%)
None	42 (85.7%)	21 (80.8%)	309 (82.6%)	101 (70.6%)	474 (79.9%)
Total	49 (100%)	26 (100%)	374 (100%)	143 (100%)	592 (100%)

Number of suicides (%) are shown

### Factors associated with suicide in adolescents

**Fig. 3:**
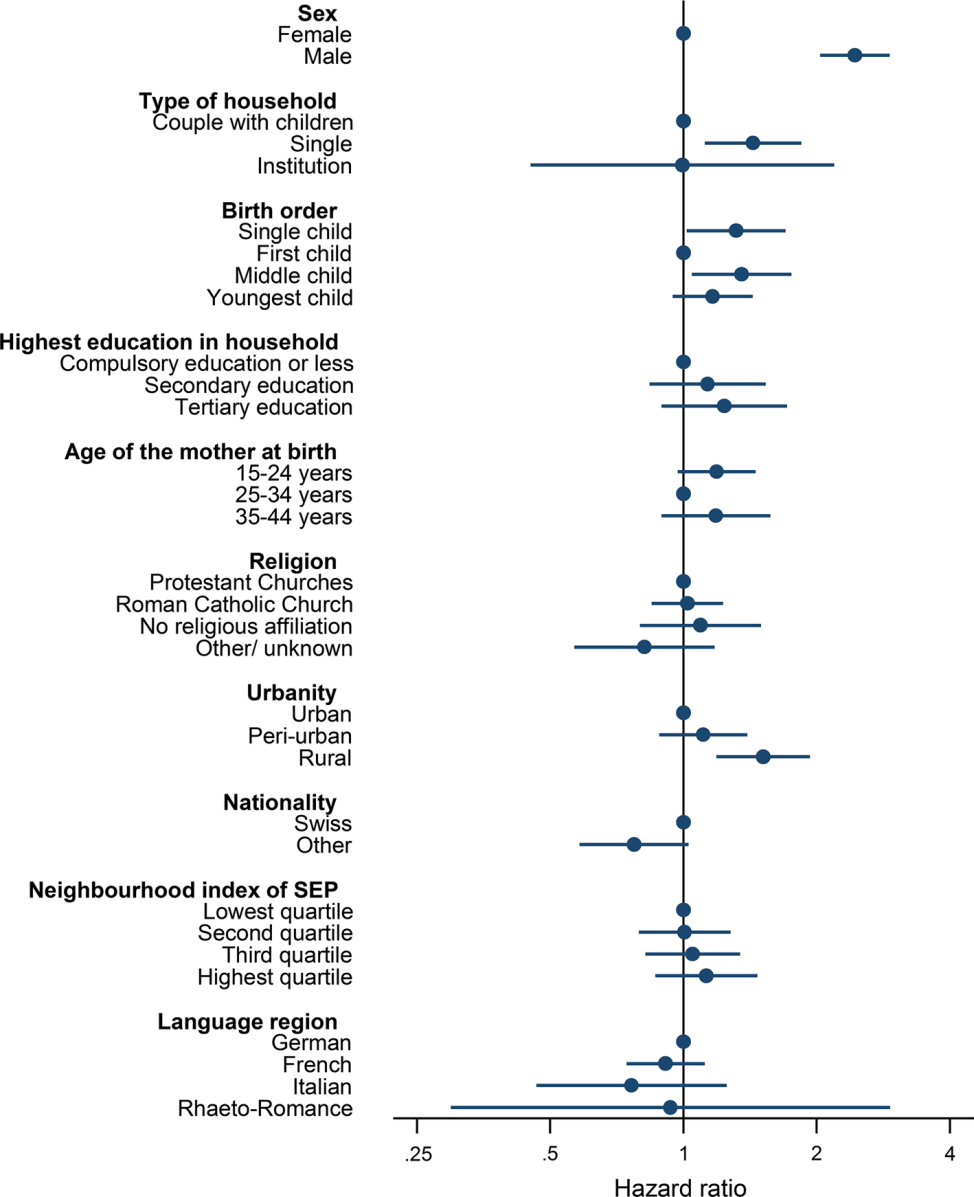
Factors associated with suicide among adolescents in Switzerland, 1991–2013: hazard ratios and 95% confidence intervals from multivariable Cox analysis. Models adjusted for sex, type of household, birth order, highest education in the household, age of mother at birth, religion, urbanity, nationality, neighbourhood index of socioeconomic position and language region

The univariable Cox models showed higher rates of suicides in boys than in girls, adolescents living with one adult, and for only children and middle-born children (Table 1). Adolescents living in households with adults with tertiary education had a higher suicide rate than those living with adults with compulsory education or less. Swiss adolescents had a higher rate than those with another passport, and the rate was higher in rural than in peri-urban and urban areas (Table 1). The results of the multivariable analysis are shown in Fig. 3. They largely confirmed the associations seen in univariable analyses: The rate was higher in boys than in girls, in single adult households than in multiple adult households, and higher in only children or middle-born children compared to firstborns. Furthermore, rates of youth suicide were higher in rural areas than in urban areas.

### Interactions with age

**Fig. 4:**
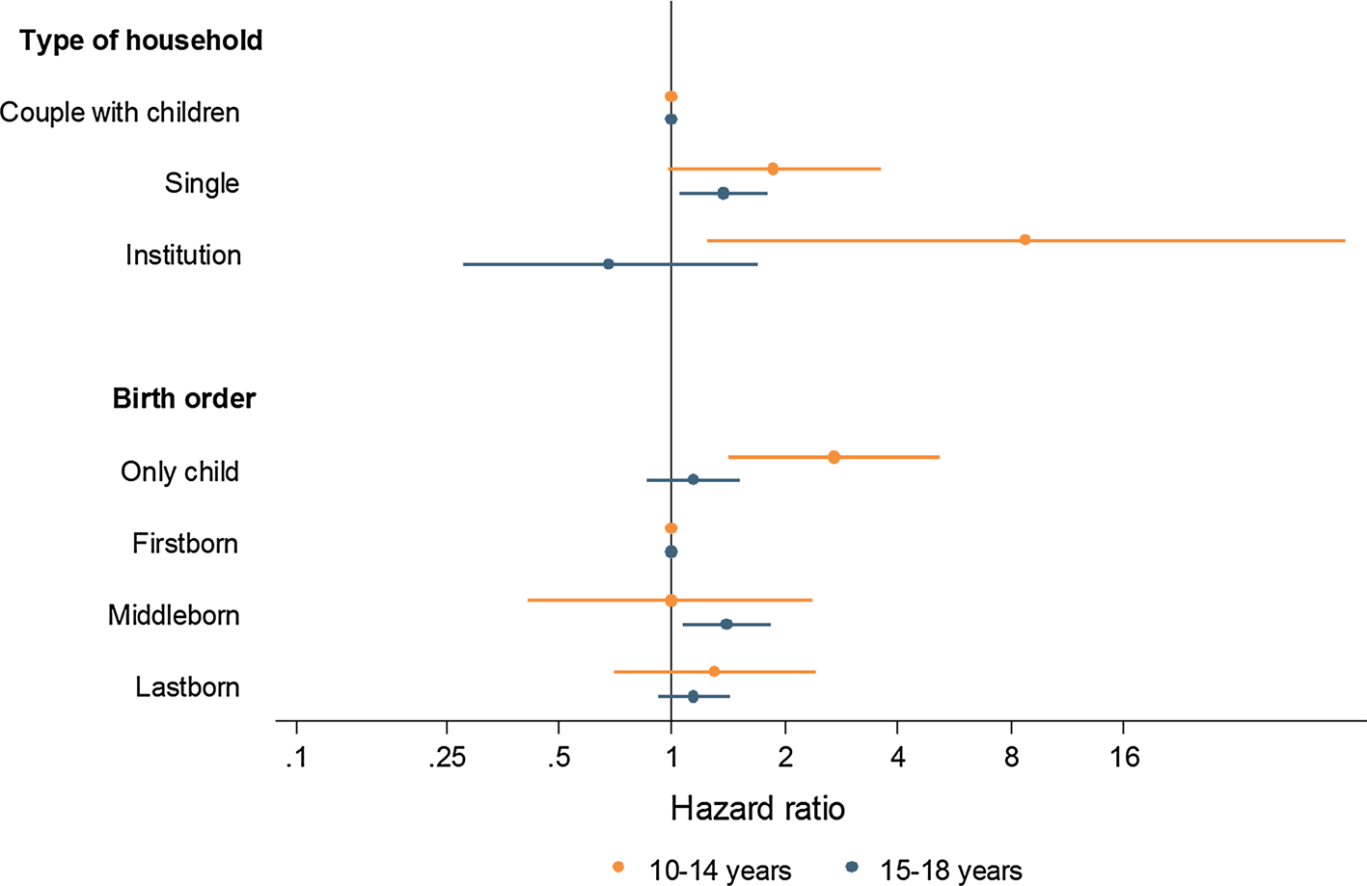
Associations of household type and birth order with suicide among adolescents in Switzerland, 1991–2013: hazard ratios and 95% confidence intervals from multivariable Cox analysis. Models adjusted for sex, type of household, birth order, highest education in the household, age of mother at birth, religion, urbanity, nationality, neighbourhood index of socioeconomic position and language region. The two variables with a statistically significant interaction with age group are shown

There were 75 suicides in age group 10–14 years and 517 suicides in age group 15–18 years (Table 1). Most adolescents (1,830,867; 76.4%) contributed person-time to both age groups. Supplementary Table 1 shows the results from multivariable analyses stratified by age group. There was evidence for an interaction with age group for two variables: type of household (*p* from test of interaction 0.032) and birth order (*p* = 0.033). Figure 4 shows the hazard ratios for these two variables. Associations between the rate of suicide and living with one adult only, or living in an institution were stronger for younger than for older adolescents. Similarly, the association with being an only child with suicide was stronger for younger adolescents. Supplementary Table 2 gives the results of all tests for interaction.

## Discussion

This longitudinal study showed that in Switzerland during the years 1991–2013 suicide in adolescents was associated with socioeconomic and demographic factors: being male, living with only one parent and living in rural areas were risk factors for suicide among youth. There was some evidence that the effect of the household composition on probability of youth suicide depended on the age group, with stronger associations in younger adolescents. Hanging was the most common method of suicide overall and in boys, whereas in girls, railway suicides were most common. Only about 20% of all adolescents dying by suicide from 1991 to 2013 had a psychiatric diagnosis recorded on the death certificate. There was no evidence for an increase or decrease over calendar time.

The rate of suicide was lower than the rate observed in Austria, where 4.57 per 100,000 adolescents from 10 to 19 years died by suicide from 2001 to 2014 [20]. Eurostat, the statistical office of the European Union, reports suicide rates only for age group 15–19 years: the rate of the 28 EU countries for the year 2013 is 4.5 per 100,000 and therefore lower than the rate in Swiss youth aged 15–18 years observed in this study [29]. The rates across countries ranged widely, from 1.29 in Greece to 21.4 in Lithuania [29]. There are only few data on trends over calendar years for suicides among adolescents. In analyses based on WHO data, the suicide rates in European adolescents decreased in boys 10–14 years old and in both genders 15–19 years old [21, 22]. In Austria the rate in 10–19 year old males decreased from 2001 to 2014, from around 10.0 to 8.0 per 100,000 person-years [20]. A slight decrease from around 2.5 to 1.5 per 100,000 person-years was also seen in girls, but it failed to reach statistical significance [20]. In England and Wales, the rates of suicides in adolescents 15–19 years old decreased between 2001 and 2010, from around 8.0 to 4.75 per 100,000 person-years, while there was no trend over time in the age group 10–14 years [19]. We found no evidence for a trend over time in suicide rates in adolescents living in Switzerland, in contrast to adults in whom the rate of suicide decreased over the last decades [30]. The methods of suicide observed confirm the results of an earlier study from Switzerland [31]. While hanging is also a common method of youth suicide in other countries [7, 13, 17, 32], railway suicides in adolescents have increased in the past years in Switzerland [31]. International comparisons emphasize the importance of the availability of suicide methods and the associated potential in prevention [32, 33].

Over 70% of the adolescents dying by suicide were boys, in line with the literature [3, 6, 8]. Mirroring the situation in adults, female adolescents have a higher rate of self-harm and suicide attempts, but a lower rate of suicide [3]. Our data confirm that the male-to-female ratio of suicide is increasing from prepubescent children to adolescents [18]. Another risk factor known from suicide in adults is living in rural areas [34–36].

Other factors are more youth-specific and often similar to risk factors for self-harm in adolescents [3]: the higher risk associated with living with one parent only is consistent with results from studies showing a higher risk for children not living in with their biological parents [6, 13]. It is unclear from our data to what extent the family constellations identified in our study, or the factors leading to these situations are causally related to the risk of suicide, or if issues such as unemployment or mental illness in parents pose a greater risk if the child has no other caregiver in the household. An earlier study found an association between parental separation and suicide, but the effect decreased when the mental health of the parents was taken into account [6].

Earlier studies found differences in the possible factors triggering suicide between younger and older adolescents: suicides in adolescents younger than 15 have often been associated with family conflicts, maltreatment or problems at school, while romantic relationships and psychiatric illness were more common risk factors in older adolescents [7, 16–18]. Brothers and sisters may be an important source of support in parent–child conflicts, which might explain the association with only children found in this study among younger adolescents.

We found a higher suicide rate in middle-born adolescents. Studies of birth-order effects on personality and psychological development have produced contradictory results [11, 37, 38]. In the context of suicide in young adults some studies showed an increasing risk with increasing number of siblings [11, 15]. In our study, less than 10% of adolescents came from families with more than three children at census.

In only few suicides, a psychiatric disorder was noted on the death certificate. The proportion of about 20% is comparable to the results of a study from Norway, although the authors restricted analyses to youths under 16 years of age [39]. In their study, the parents of those who died by suicide more often reported mental health problems in their children than parents of accident victims, but there were no significant differences in the Kiddie-SADS criteria for any psychiatric diagnoses [39]. Conversely, studies from the US found that 80–90% of all suicides in adolescents were associated with psychiatric morbidity [4, 6]. In Australia, 50% of children aged 10–14 years and 57% of adolescents aged 15–17 years who died by suicide had a mental disorder [40]. Whereas it is likely that the information on the death certificate underestimates the prevalence of mental illness among youth dying by suicide, the post hoc psychological autopsy approach will overestimate the prevalence of psychiatric morbidity [41]. In the USA and Canada studies based on coroner data also found a considerably higher proportion of adolescent suicides with mental disorders [42, 43]. It is unclear whether Swiss adolescents dying by suicide are suffering less often from psychiatric disorders or if they are underdiagnosed. Of note, a recent report on the care of mentally ill persons in Switzerland identified a shortage of resources in the psychiatric care for children and adolescents [44].

An important strength of our study is its national coverage, and the detailed socioeconomic and demographic data available both at the individual and household level. This study was, however, based on routine data collected at the census and on the death certificate, and we could not investigate the importance of mental illness in the adolescents or their parents in any detail. We also had no information on the relation of the adolescents with their parents, teachers and schoolmates. All our data on sociodemographic factors are based on the census 1990 and 2000. Therefore some factors as type of household, highest education in household or birth order might have changed during the study period. In addition, we could only identify the parents of the adolescents if they were living in the same household with the child at least at the time of one census.

Misclassification of suicide as accidental or other unnatural death [45, 46] is another limitation of studies based on death certificate data. For example, a survey among medical examiners in the USA revealed potential for misclassification particularly in suicides of youth [47]. Of note, suicide notes and data on past suicidal behaviour or diagnosed mental disorders are important in decision-making processes of medical examiners in the determination of child suicide [47]. Unfortunately, we did not have any information on suicide notes and suicidal behaviour, and only limited information on diagnosed mental disorders from the death certificates. However, systematic misclassification is unlikely in Switzerland because the family and the communal authorities do not receive a copy of the death certificate, and death registration is anonymous.

In conclusion, further research is needed to better understand the complex effects of distal and proximal risk factors and protective factors on suicide in adolescents. Mental health problems of children or their parents, parent–child conflicts and mobbing have been shown to be risk factors for suicide [7, 14], with a dose–response association [9]. Familial and sociodemographic factors such as the composition of the household may reflect the level of social support available to the adolescent, which in turn may mediate the resilience of adolescents to deal with conflicts and stressors. These factors should therefore be considered when designing preventive interventions in adolescents at risk.

## Electronic supplementary material

Below is the link to the electronic supplementary material.

Supplementary material 1 (DOCX 21 kb)

Supplementary material 2 (DOCX 14 kb)

## References

[1] WHO (2014) Preventing suicide: a global imperative. http://www.who.int/mental_health/suicide-prevention/world_report_2014/en/. Accessed 28 Jan 2015

[2] Pelkonen M, Marttunen M (2003). Child and adolescent suicide: epidemiology, risk factors, and approaches to prevention. Paediatr Drugs.

[3] Hawton K, Saunders KE, O’Connor RC (2012). Self-harm and suicide in adolescents. Lancet.

[4] Evans E, Hawton K, Rodham K (2004). Factors associated with suicidal phenomena in adolescents: a systematic review of population-based studies. Clin Psychol Rev.

[5] Evans E, Hawton K, Rodham K, Deeks J (2005). The prevalence of suicidal phenomena in adolescents: a systematic review of population-based studies. Suicide Life Threat Behav.

[6] Gould MS, Greenberg T, Velting DM, Shaffer D (2003). Youth suicide risk and preventive interventions: a review of the past 10 years. J Am Acad Child Adolesc Psychiatry.

[7] Soole R, Kolves K, De Leo D (2015). Suicide in children: a systematic review. Arch Suicide Res.

[8] Rhodes AE, Boyle MH, Bridge JA, Sinyor M, Links PS, Tonmyr L, Skinner R, Bethell JM, Carlisle C, Goodday S, Hottes TS, Newton A, Bennett K, Sundar P, Cheung AH, Szatmari P (2014). Antecedents and sex/gender differences in youth suicidal behavior. World J Psychiatry.

[9] Bjorkenstam C, Kosidou K, Bjorkenstam E (2017). Childhood adversity and risk of suicide: cohort study of 548 721 adolescents and young adults in Sweden. BMJ.

[10] Bridge JA, Goldstein TR, Brent DA (2006). Adolescent suicide and suicidal behavior. J Child Psychol Psychiatry.

[11] Riordan DV, Selvaraj S, Stark C, Gilbert JS (2006). Perinatal circumstances and risk of offspring suicide. Birth cohort study. Br J Psychiatry.

[12] Mittendorfer-Rutz E, Rasmussen F, Wasserman D (2004). Restricted fetal growth and adverse maternal psychosocial and socioeconomic conditions as risk factors for suicidal behaviour of offspring: a cohort study. Lancet.

[13] Beautrais AL (2001). Child and young adolescent suicide in New Zealand. Aust N Z J Psychiatry.

[14] Brent DA, Baugher M, Bridge J, Chen T, Chiappetta L (1999). Age- and sex-related risk factors for adolescent suicide. J Am Acad Child Adolesc Psychiatry.

[15] Bjorngaard JH, Bjerkeset O, Vatten L, Janszky I, Gunnell D, Romundstad P (2013). Maternal age at child birth, birth order, and suicide at a young age: a sibling comparison. Am J Epidemiol.

[16] Coskun M, Zoroglu S, Ghaziuddin N (2012). Suicide rates among Turkish and American youth: a cross-cultural comparison. Arch Suicide Res.

[17] Groholt B, Ekeberg O (2003). Suicide in young people under 15 years: problems of classification. Nord J Psychiatry.

[18] Peyre H, Hoertel N, Stordeur C, Lebeau G, Blanco C, McMahon K, Basmaci R, Lemogne C, Limosin F, Delorme R (2017). Contributing factors and mental health outcomes of first suicide attempt during childhood and adolescence: results from a nationally representative study. J Clin Psychiatry.

[19] Windfuhr K, While D, Hunt IM, Shaw J, Appleby L, Kapur N (2013). Suicide and accidental deaths in children and adolescents in England and Wales, 2001–2010. Arch Dis Child.

[20] Laido Z, Voracek M, Till B, Pietschnig J, Eisenwort B, Dervic K, Sonneck G, Niederkrotenthaler T (2016). Epidemiology of suicide among children and adolescents in Austria, 2001–2014. Wien Klin Wochenschr.

[21] Kolves K, De Leo D (2014). Suicide rates in children aged 10–14 years worldwide: changes in the past two decades. Br J Psychiatry.

[22] Kolves K, De Leo D (2016). Adolescent suicide rates between 1990 and 2009: analysis of age group 15–19 years worldwide. J Adolesc Health.

[23] Bopp M, Spoerri A, Zwahlen M, Gutzwiller F, Paccaud F, Braun-Fahrlander C, Rougemont A, Egger M (2009). Cohort profile: the Swiss National Cohort—a longitudinal study of 6.8 million people. Int J Epidemiol.

[24] Spoerri A, Zwahlen M, Egger M, Bopp M (2010). The Swiss National Cohort: a unique database for national and international researchers. Int J Public Health.

[25] Schmidlin K, Clough-Gorr KM, Spoerri A, Egger M, Zwahlen M (2013). Impact of unlinked deaths and coding changes on mortality trends in the Swiss National Cohort. BMC Med Inform Decis Mak.

[26] Renaud A (2004) Methodology report—coverage estimation for the Swiss population census 2000. Swiss Federal Statistical Office, Neuchâtel, pp 1–147. Available from https://www.bfs.admin.ch/bfsstatic/dam/assets/341896/master. Accessed 27 June 2017

[27] Panczak R, Galobardes B, Voorpostel M, Spoerri A, Zwahlen M, Egger M (2012). A Swiss neighbourhood index of socioeconomic position: development and association with mortality. J Epidemiol Commun Health.

[28] Bertolote JM, Fleischmann A (2002). Suicide and psychiatric diagnosis: a worldwide perspective. World Psychiatry.

[29] Eurostat General and regional statistics. Suicide death rate, by age group. Statistical Office of the European Communities, Luxembourg. Available form http://ec.europa.eu/eurostat/tgm/table.do?tab=table&init=1&language=en&pcode=tsdph240&plugin=1. Accessed 27 June 2017

[30] Hepp U, Ring M, Frei A, Rössler W, Schnyder U, Ajdacic-Gross V (2010). Suicide trends diverge by method: Swiss suicide rates 1969–2005. Eur Psychiatry.

[31] Hepp U, Stulz N, Unger-Koppel J, Ajdacic-Gross V (2012). Methods of suicide used by children and adolescents. Eur Child Adolesc Psychiatry.

[32] Kolves K, de Leo D (2017). Suicide methods in children and adolescents. Eur Child Adolesc Psychiatry.

[33] Ajdacic-Gross V, Weiss MG, Ring M, Hepp U, Bopp M, Gutzwiller F, Rossler W (2008). Methods of suicide: international suicide patterns derived from the WHO mortality database. Bull World Health Organ.

[34] Steck N, Egger M, Zwahlen M, Swiss National C (2016). Assisted and unassisted suicide in men and women: longitudinal study of the Swiss population. Br J Psychiatry.

[35] Middleton N, Gunnell D, Frankel S, Whitley E, Dorling D (2003). Urban–rural differences in suicide trends in young adults: England and Wales, 1981-1998. Soc Sci Med (1982).

[36] Hirsch JK (2006). A review of the literature on rural suicide: risk and protective factors, incidence, and prevention. Crisis.

[37] Ernst C, Angst J (1983). Birth order: its influence on personality.

[38] Damian RI, Roberts BW (2015). Settling the debate on birth order and personality. Proc Natl Acad Sci USA.

[39] Freuchen A, Kjelsberg E, Lundervold AJ, Groholt B (2012). Differences between children and adolescents who commit suicide and their peers: a psychological autopsy of suicide victims compared to accident victims and a community sample. Child Adolesc Psychiatry Ment Health.

[40] Soole R, Kolves K, De Leo D (2014). Factors related to childhood suicides: analysis of the Queensland Child Death Register. Crisis.

[41] Cavanagh JT, Carson AJ, Sharpe M, Lawrie SM (2003). Psychological autopsy studies of suicide: a systematic review. Psychol Med.

[42] Sinyor M, Schaffer A, Cheung AH (2014). An observational study of bullying as a contributing factor in youth suicide in Toronto. Can J Psychiatry.

[43] Karch DL, Logan J, McDaniel DD, Floyd CF, Vagi KJ (2013). Precipitating circumstances of suicide among youth aged 10–17 years by sex: data from the National Violent Death Reporting System, 16 states, 2005–2008. J Adolesc Health.

[44] Stocker D, Stettler P, Jäggi J, Bischof S, Guggenbühl T, Abrassart A, Künzi K (2016) Versorgungssituation psychisch erkrankter Personen in der Schweiz. Büro für arbeits- und sozialpolitische Studien BASS

[45] Tollefsen IM, Hem E, Ekeberg O (2012). The reliability of suicide statistics: a systematic review. BMC Psychiatry.

[46] Palmer BS, Bennewith O, Simkin S, Cooper J, Hawton K, Kapur N, Gunnell D (2015). Factors influencing coroners’ verdicts: an analysis of verdicts given in 12 coroners’ districts to researcher-defined suicides in England in 2005. J Public Health (Oxford England).

[47] Crepeau-Hobson F (2010). The psychological autopsy and determination of child suicides: a survey of medical examiners. Arch Suicide Res.

