# Supplementary material for: Suicide in adolescents: findings from the Swiss National cohort
Source: Eur Child Adolesc Psychiatry. 2017 Jun 29;27(1):47–56. doi: 10.1007/s00787-017-1019-6 (PMC5799333; doi:10.1007/s00787-017-1019-6)
Supplement: Supplementary file 1 — Supplementary material 1 (DOCX 21 kb) [file 787_2017_1019_MOESM1_ESM.docx]

**Supplementary Table 1. Crude rates of suicides among adolescents in Switzerland 1991-2030 cand results from the multivariable Cox model, by age group.**

|  | | **Age group** | | | | | | | |
| --- | --- | --- | --- | --- | --- | --- | --- | --- | --- |
|  | | **10-14 years old** | | | | **15-18 years old** | | | |
| Study population |  | 2,014,971 | | | | 2,211,573 | | | |
| No of suicides |  | 75 | | | | 517 | | | |
|  | | **Crude rate per 100,00 (95%CI)** | | **Hazard ratio from multivariable Cox model (95%CI)** | | **Crude rate per 100,00 (95%CI)** | | **Hazard ratio from multivariable Cox model (95%CI)** | |
| Overall |  | 0.86 | (0.68-1.1) |  |  | 7.00 | (6.43-7.64) |  |  |
| Sex | Female | 0.61 | (0.42-0.90) | 1 |  | 3.98 | (3.38-4.69) | 1 |  |
|  | Male | 1.09 | (0.83-1.45) | 1.74 | (1.08-2.81) | 9.88 | (8.93-10.94) | 2.57 | (2.11-3.13) |
| Type of household | Couple w. children | 0.74 | (0.57-0.95) | 1 |  | 6.74 | (6.13-7.41) | 1 |  |
|  | Single w. children | 1.80 | (1.05-3.11) | 1.88 | (0.98-3.62) | 8.86 | (7.00-11.22) | 1.37 | (1.04-1.80) |
|  | Insitution | 3.21 | (1.04-10.0) | 8.79 | (1.24-62.27) | 5.17 | (2.46-10.84) | 0.68 | (0.28-1.69) |
|  | Other | 1.92 | (0.27-13.62) |  |  | 13.30 | (7.16-24.72) |  |  |
| Birth order | Only child | 1.54 | (1.00-2.36) | 2.71 | (1.42-5.18) | 7.25 | (5.79-9.07) | 1.14 | (0.86-1.52) |
|  | Firstborn | 0.62 | (0.39-0.98) | 1 |  | 6.10 | (5.19-7.15) | 1 |  |
|  | Middleborn | 0.64 | (0.30-1.34) | 0.99 | (0.41-2.39) | 8.50 | (6.84-10.57) | 1.40 | (1.06-1.83) |
|  | Lastborn | 0.78 | (0.52-1.18) | 1.30 | (0.70-2.42) | 6.98 | (6.00-8.12) | 1.15 | (0.92-1.43) |
|  | Unknown | 1.38 | (0.62-3.07) |  |  | 8.13 | (6.03-10.96) |  |  |
| Highest education in household | Compulsory or less | 1.02 | (0.55-1.89) | 1 |  | 5.23 | (3.95-6.92) | 1 |  |
|  | Secondary | 0.81 | (0.58-1.13) | 0.70 | (0.32-1.50) | 7.14 | (6.32-8.07) | 1.22 | (0.88-1.70) |
|  | Tertiary | 0.80 | (0.53-1.20) | 0.83 | (0.35-1.94) | 7.44 | (6.40-8.65) | 1.32 | (0.89-1.63) |
|  | Unknown | 0.44 | (0.06-3.13) | 0.97 | (0.22-4.21) | 7.35 | (5.43-9.95) | 1.41 | (0.81-2.50) |
| Age of mother at birth (in years) | 15-24 | 1.29 | (0.86-1.95) | 1.95 | (1.14-3.34) | 6.94 | (5.80-8.31) | 1.09 | (0.88-1.36) |
|  | 25-34 | 0.67 | (0.49-0.92) | 1 |  | 6.79 | (6.05-7.57) | 1 |  |
|  | 35-44 | 0.91 | (0.46-1.83) | 1.20 | (0.55-2.59) | 7.99 | (6.05-10.54) | 1.20 | (0.86-1.63) |
|  | Unknown | 1.37 | (0.62-3.06) |  |  | 8.07 | (5.99-10.88) |  |  |
| Marital status of mother at census | Single | 1.01 | (0.25-4.06) |  |  | 9.42 | (5.07-17.52) |  |  |
|  | Married | 0.74 | (0.57-0.96) |  |  | 6.78 | (6.16-7.46) |  |  |
|  | Widowed | 3.61 | (0.90-14.4) |  |  | 7.60 | (3.42-16.93) |  |  |
|  | Divorced | 2.16 | (1.08-4.32) |  |  | 8.20 | (5.92-11.37) |  |  |
|  | Unknown | 1.38 | (0.62-3.07) |  |  | 8.13 | (6.03-10.96) |  |  |
| Age of father at birth (in years) | 15-24 | 0.79 | (0.33-1.89) |  |  | 5.29 | (3.76-7.44) |  |  |
|  | 25-34 | 0.75 | (0.54-1.03) |  |  | 7.08 | (6.31-7.94) |  |  |
|  | 35-44 | 0.68 | (0.79-0.33) |  |  | 6.47 | (5.21-8.04) |  |  |
|  | ≥45 | 0.65 | (0.09-4.58) |  |  | 6.25 | (2.98-13.11) |  |  |
|  | Unknown | 1.73 | (1.10-2.71) |  |  | 8.23 | (6.78-10.00) |  |  |
| Maritals status of father at census | Single | 0.95 | (0.13-6.73) |  |  | 5.68 | (1.83-17.62) |  |  |
|  | Married | 0.74 | (0.57-0.96) |  |  | 6.71 | (6.08-7.40) |  |  |
|  | Widowed | - |  |  |  | 4.93 | (0.69-35.02) |  |  |
|  | Divorced | - |  |  |  | 11.63 | (6.05-22.35) |  |  |
|  | Unknown | 1.73 | (1.10-2.71) |  |  | 8.25 | (6.79-10.01) |  |  |
| Religion | Protestant | 1.10 | (0.78-1.54) | 1 |  | 7.31 | (6.36-8.41) | 1 |  |
|  | Catholic | 0.72 | (0.50-1.05) | 0.67 | (0.39-1.15) | 7.26 | (6.39-8.25) | 1.09 | (0.88-1.36) |
|  | No affiliation | 0.77 | (0.34-1.70) | 0.73 | (0.30-1.78) | 7.58 | (5.64-10.19) | 1.20 | (0.89-1.63) |
|  | Other/Unknown | 0.71 | (0.36-1.43) | 0.84 | (0.33-2.11) | 4.72 | (3.47-6.41) | 0.50 | (0.30-0.84) |
| Nationality | Swiss | 0.94 | (0.74-1.20) | 1 |  | 7.52 | (6.85-8.26) | 1 |  |
|  | Other | 0.55 | (0.30-1.03) | 0.53 | (0.23-1.22) | 5.01 | (4.00-6.28) | 0.82 | (0.60-1.10) |
| Language Region | German | 0.94 | (0.73-1.21) | 1 |  | 7.17 | (6.49-7.93) | 1 |  |
|  | French | 0.58 | (0.33-1.02) | 0.66 | (0.35-1.24) | 6.86 | (5.74-8.22) | 0.95 | (0.77-1.18) |
|  | Italian | 1.20 | (0.45-3.20) | 1.52 | (0.53-4.38) | 4.50 | (2.61-7.75) | 0.66 | (0.38-1.17) |
|  | Rhaeto-Romance | - | - |  |  | 9.40 | (3.03-29.16) |  |  |
| Urbanisation | Urban | 0.84 | (0.52-1.35) | 1 |  | 5.58 | (4.56-6.82) | 1 |  |
|  | Peri-Urban | 0.75 | (0.53-1.08) | 0.87 | (0.47-1.60) | 6.54 | (5.73-7.46) | 1.15 | (0.90-1.48) |
|  | Rural | 1.03 | (0.71-1.49) | 1.34 | 0.70-2.56) | 8.75 | (7.62-10.04) | 1.55 | (1.19-2.02) |
| Neighbourhood Index of SEP (quartiles) | Lowest | 0.75 | (0.46-1.22) | 1 |  | 6.83 | (5.72-8.15) | 1 |  |
|  | Second | 0.94 | (0.60-1.45) | 1.20 | (0.62-2.34) | 6.91 | (5.80-8.24) | 0.98 | (0.76-1.27) |
|  | Third | 0.89 | (0.56-1.39) | 1.16 | (0.58-2.34) | 7.15 | (6.02-8.50) | 1.03 | (0.79-1.35) |
|  | Highest | 0.84 | (0.53-1.33) | 1.21 | (0.57-2.61) | 7.26 | (6.13-8.61) | 1.11 | (0.84-1.48) |
|  | Unknown | 1.18 | (0.30-4.74) |  |  | 5.38 | (2.69-10.76) |  |  |

SEP, socio-economic position
