# Supplementary material for: Suicide in adolescents: findings from the Swiss National cohort
Source: Eur Child Adolesc Psychiatry. 2017 Jun 29;27(1):47–56. doi: 10.1007/s00787-017-1019-6 (PMC5799333; doi:10.1007/s00787-017-1019-6)
Supplement: Supplementary file 2 — Supplementary material 2 (DOCX 14 kb) [file 787_2017_1019_MOESM2_ESM.docx]

**Supplementary Table 2. Results from tests of interactions between risk factors for suicide among adolescents in Switzerland 1991-2013 and age group (10-14 years or 15-18 years).**

| **Variable** | **Chi-squared value** | **Degrees of freedom** | **P** |
| --- | --- | --- | --- |
| Sex | 1.99 | 1 | 0.1583 |
| Type of household | 6.89 | 2 | 0.0320 |
| Birth order | 10.52 | 4 | 0.0325 |
| Education | 2.78 | 3 | 0.4262 |
| Age of mother | 4.83 | 3 | 0.1835 |
| Religion | 3.09 | 3 | 0.3778 |
| Nationality | 0.05 | 1 | 0.8246 |
| Language region | 3.82 | 2 | 0.1478 |
| Urbanisation | 1.01 | 2 | 0.6033 |
| Neighbourhood index of SEP | 0.47 | 4 | 0.9767 |
